# Cystathionine β-synthase affects organization of cytoskeleton and modulates carcinogenesis in colorectal carcinoma cells

**DOI:** 10.3389/fonc.2023.1178021

**Published:** 2023-07-07

**Authors:** Veronika Liskova, Barbora Chovancova, Petr Babula, Ingeborg Rezuchova, Kristina Ploth Pavlov, Miroslava Matuskova, Olga Krizanova

**Affiliations:** ^1^Institute of Clinical and Translational Research, Biomedical Research Center, Slovak Academy of Sciences, Bratislava, Slovakia; ^2^Department of Physiology, Faculty of Medicine, Masaryk University, Brno, Czechia; ^3^Institute of Virology, Biomedical Research Center, Slovak Academy of Sciences, Bratislava, Slovakia; ^4^Cancer Research Institute, Biomedical Research Center, Slovak Academy of Sciences, Bratislava, Slovakia

**Keywords:** cystathionine beta-synthase, cytoskeleton, β-tubulin, colorectal carcinoma cells, xenografts

## Abstract

**Background:**

Cystathionine β-synthase (CBS), one of three enzymes that endogenously produce hydrogen sulfide, is extensively studied for its relevance in the cells of various tumors. In our previous work, we observed that the immunofluorescence pattern of CBS is very similar to that of tubulin and actin. Therefore, we focused on the potential interaction of CBS with cytoskeletal proteins β-actin and β-tubulin and the functional relevance of the potential interaction of these proteins in colorectal carcinoma cell lines.

**Methods:**

To study the potential interaction of CBS with cytoskeletal proteins and its functional consequences, a CBS-knockout DLD1 (DLDx) cell line was established by using the CRISPR/Cas9 gene editing method. The interaction of the selected cytoskeletal protein with CBS was studied by immunoprecipitation, Western blot analysis, immunofluorescence, and proximity ligation assay. The functional consequences were studied by proliferation and migration assays and by generation of xenografts in SCID/bg mice.

**Results:**

We have found that CBS, an enzyme that endogenously produces H2S, binds to cytoskeletal β-tubulin and, to a lesser extent, also to β-actin in colorectal carcinoma-derived cells. When CBS was knocked out by the CRISPR/Cas9 technique (DLDx), we observed a de-arranged cytoskeleton compared to the unmodified DLD1 cell line. Treatment of these cells with a slow sulfide donor GYY4137 resulted in normal organization of the cytoskeleton, thus pointing to the role of CBS in microtubule dynamics. To evaluate the physiological importance of this observation, both DLD1 and DLDx cells were injected into SCID/bg mice, and the size and mass of the developed xenografts were evaluated. Significantly larger tumors developed from DLDx compared to the DLD1 cells, which correlated with the increased proliferation of these cells.

**Conclusions:**

Taken together, in colorectal cancer DLD1 cells, CBS binds to the cytoskeleton, modulates microtubule dynamics, and thus affects the proliferation and migration in the colorectal carcinoma stable cell line.

## Introduction

Cystathionine β-synthase (CBS) is one of three enzymes that endogenously produce hydrogen sulfide (H_2_S). H_2_S is a relatively novel gaseous transmitter, to which several regulatory roles have already been attributed. CBS is localized both in the cytoplasm and mitochondria (1) and is regulated by a variety of physiological factors, e.g., 1,25-dihydroxyvitamin D3, hypoxia-inducible factors, and estradiol-17β (for a review, see 2), depending on the cell type. Using cryo-electron microscopy, McCorvie and co-workers (3) have shown that human CBS reveals a filamentous architecture. This full-length enzyme polymerizes as an active filament that changes conformation due to S-adenosyl-L-methionine. Interactions due to filamentation result in a shift of the residues 417-423 on the C-terminal regulatory domain. The physiological role of CBS is still not completely clear, although its detrimental effect on the cardiovascular system is evident (4). Permanent CBS gene knockdown in immortalized human adipose-derived mesenchymal stem cells promoted a cellular senescence phenotype with increased adipogenic potential and excessive lipid storage (5). The regulation of immune and inflammatory responses by CBS has also been described (6).

The role of CBS in carcinogenesis has been studied on various types of tumors by several laboratories using different approaches. Increased levels of CBS were detected in colon/colorectal (7, 8), breast, and prostate cancer (9). This enzyme was also found to be increased in thyroid malignancies (10). In ovarian cancer cells, CBS regulates bioenergetics by regulating mitochondrial ROS production, oxygen consumption, and ATP generation (11). In breast cancer, CBS-derived H_2_S might play a role in the protection of breast cancer cells against activated macrophages (12). Inhibition of CBS can improve ovarian cancer treatment as well (13). In colon cancer-derived epithelial cell lines, a role for endogenous H_2_S in tumor angiogenesis has been shown (7). However, decreased CBS expression was determined in clear cell renal cell carcinoma that was spontaneously hypoxic compared to the matched controls, and this decrease was dependent on the grade of tumor (14). A question remains as to whether higher CBS levels in tumors together with overexpression of other H_2_S-synthesizing enzymes could be due to a lower amount of H_2_S in tumors, as suggested by Dongsoo et al. (15). A bell-shaped model has been proposed to explain the role of H_2_S in cancer development. Specifically, endogenous H_2_S or a relatively low level of exogenous H_2_S may exhibit a pro-cancer effect, whereas exposure to H_2_S at a higher amount or for a long period may lead to cancer cell death (16). In favor of this hypothesis might be the fact that the slow sulfide donor GYY4137 can induce apoptosis in stable cancer cell lines *in vitro* and also in xenografts induced in immunodeficient mice treated with GYY4137 (17, 18).

CBS has been shown to be localized mainly in cytosol and to a lesser extend in mitochondria (19, 20). However, we have recently shown a specific CBS pattern by immunofluorescent staining (14), similar to staining of the cytoskeleton. Thus, we produced a hypothesis that CBS might bind to cytoskeletal proteins.

Microtubules are major cytoskeletal components in the eukaryotic cells. Microtubules and microtubule dynamics are involved in several important activities, such as maintenance of cell shape and cell motility, cell division and accurate chromosome segregation during mitosis, general intracellular communication, and intracellular tracking of macromolecules and organelles in the interphase. In cancer cells, a high expression of several β-tubulin isotypes correlates with aggressive clinical behavior, chemotherapy drug resistance, and poor prognosis (21). Cytotoxic action of microtubule-targeting drugs (such as taxanes or vinca alkaloids) is based on the variations of microtubule dynamics. Modulation of microtubules can significantly affect the fate of cells. Chaudhuri and co-workers (22) have shown that tubulin disulfides may play a role in tubulin folding and that thiol–disulfide exchange in tubulin could be a key regulator in microtubule assembly and dynamics of tubulin *in vivo*. Under physiological conditions, tubulins are sulfhydrated, which affects microtubule assembly (23). Hosono et al. (24) showed the effect of diallyl trisulfide (DATS) on the modification of cysteines Cys-12β and Cys-354β in β-tubulin, which is assumed to be a cause for the disruption of microtubule network formation. Nevertheless, microtubule assembly is dependent on a variety of other factors (e.g., acetylation, GTP, and phosphorylation), and a mutual interplay of all these factors should be also considered.

Based on the current knowledge on CBS and also on our previous experiments, we aimed to study the potential interaction of CBS and some cytoskeletal proteins, i.e., β-actin and β-tubulin (this type of tubulin has binding sites for taxanes and vinca alkaloids, etc. (25)). Since the cytoskeleton is crucially involved in carcinogenesis and is a target of some groups of chemotherapeutics, cancer cell growth and proliferation due to the potential interaction with CBS was studied as well.

## Materials and methods

### Cell cultivation and treatments

Experiments were performed on colorectal carcinoma cell lines DLD1 (CCL-221, ATCC, Sigma-Aldrich, St. Louis, MO, USA) and HCT116 (CCL-247, ATCC, Sigma-Aldrich, St. Louis, MO, USA) derived from clear cell renal cell carcinoma (ccRCC) (ECACC, 03112702, Sigma-Aldrich, St. Louis, MO, USA) and a non-cancerous cell line derived from EA.hy926 epithelial cells (ATCC, CRL-2922TM). The cell lines were cultured in an RPMI medium (Sigma-Aldrich, St. Louis, MO, USA) or Dulbecco’s minimal essential medium (DMEM, Sigma-Aldrich, St. Louis, MO, USA) with high glucose (4.5 g/L) and L-glutamine (300 μg/mL), supplemented with 10% fetal bovine serum (Sigma-Aldrich, St. Louis, MO, USA) and a penicillin/streptomycin mixture (Calbiochem, San Diego, CA, USA, penicillin 100U/mL, streptomycin 100 μg/mL). The cells were cultured in a water-saturated atmosphere at 37°C and 5% CO_2_. The experiments were performed with the cells in passage 5-15, and they were tested once a week for mycoplasma by double PCR. The cells were treated with paclitaxel (PTX, Selleckchem, Pittsburgh, PA, USA, 20 nmol/L), vincristine sulfate salt (vin, Sigma-Aldrich, St. Louis, MO, USA, 100 nmol/L), and a slow-releasing sulfide donor GYY4137 (GYY, Cayman Chemical, Ann Arbor, MI, USA, 10 µmol/L) (18, 26, 27) for 24 h.

### Generation of CBS-knockout DLD1 cell line

The CBS-knockout DLD1 cell line, hereafter referred to as DLDx, was established by using the CRISPR/Cas9 (CRISPR (clustered, regularly interspaced, short palindromic repeats)/Cas9 (CRISPR-associated protein 9)) gene editing method. The CBS CRISPR guide RNA sequences (GATTTCGTTCTTCAGCCGCC and TGTGCCCTCAGGGATCGGGC) were designed by the laboratory of Feng Zhang at the Broad Institute in order to efficiently target the CBS gene with minimal risk of off-target Cas9 binding elsewhere in the genome (28, 29). The lentiviral transfer plasmids lentiCRISPRv2_CBS-1 and lentiCRISPRv2_CBS-3 (GenScript, Leiden, Netherlands) contained a lentiCRISPRv2 backbone and single above-mentioned oligo cloned into the single-guide RNA (sgRNA) scaffold. To produce the lentiviral particles, the transfer plasmids lentiCRISPRv2_CBS-1 or lentiCRISPRv2_CBS-3 were co-transfected into HEK293T cells with the packaging plasmids pMD2.G (Addgene, Watertown, MA, USA) and psPAX2 (Addgene, Watertown, MA, USA). The virus-containing medium was collected after 48, 60, and 72 h and passed through a 0.45 μm low protein-binding filter. Lentiviruses were concentrated using PEG-it (System Biosciences, Palo Alto, CA, USA) and sedimented by centrifugation (1500 × g, 4°C for 30 min). As a positive control to monitor transduction efficiency, CRISPR-lenti human EMX1 positive control transduction particles (CRISPR11V-1EA, Sigma-Aldrich, St. Louis, MO, USA) were used. Similarly, as a negative control, CRISPR-lenti non-targeting control transduction particles (CRISPR12V-1EA, Sigma-Aldrich, St. Louis, MO, USA) were used. This control includes a guide RNA sequence that does not target known human, mouse, and rat genes. DLD1 cells, plated the day before at a density of 0.25×10^5^ cells per 6 cm plate, were infected with each lentivirus or their combination. DLD1 cells transduced by control lentivirus particles were called DLD-PC (positive control) and DLD-NC (negative control). Twenty-four hours after transduction, the cells were selected by puromycin (Puromycin, InvivoGen, USA), and then, the CBS protein knockout was confirmed by immunofluorescence (IF) and Western blot analysis (WB).

### Immunofluorescence

Cells grown on glass coverslips were fixed in ice-cold methanol, as described previously (18). Non-specific binding was blocked by incubation with phosphate-buffered saline (PBS) containing 3% bovine serum albumin (BSA, Sigma-Aldrich, St. Louis, MO, USA) for 60 min at room temperature. The cells were then incubated with primary antibodies diluted in PBS with 1% BSA (PBS-BSA) for 1 h at 37°C. The antibodies specific to human CBS (1:100 dilution, AP6959c, Abgent, San Diego, CA, USA), β-tubulin (1:1000 dilution, ab231082, Abcam, Cambridge, UK), and β-actin (1:250 dilution, ab6276, Abcam, Cambridge, UK) were used. Afterwards, the cells were washed four times with PBS with 0.02% TWEEN (Sigma-Aldrich, St. Louis, MO, USA) for 10 min, incubated with Alexa Fluor-594 goat anti-mouse/anti-rabbit (1:1000 dilution, Thermo Fisher Scientific, Waltham, MA, USA) or IgG Alexa Fluor-488 donkey anti-rabbit IgG (1:1000 dilution, Thermo Fisher Scientific, Waltham, MA, USA) in PBS-BSA for 1 h at 37°C, and they were washed as described previously. Finally, coverslips were mounted onto the slides in a mounting medium with a blue-fluorescent DNA stain 4′.6-diamidino-2-phenylindole (DAPI, Sigma-Aldrich, St. Louis, MO, USA). The cells were visualized by epifluorescence microscopy using Nikon Eclipse Ti-S/L100 (Nikon, Japan), and NIS elements software (Nikon, Tokyo, Japan) was used to process the images and evaluate the resultant pictures.

### Proximity ligation assay

The proximity ligation assay (PLA) was used for *in situ* detection of the co-localization between CBS and β-tubulin or β-actin. The assay was performed in a humid chamber at 37°C according to the manufacturer’s instructions (Olink Bioscience, Uppsala, Sweden). The cells were seeded on glass coverslips and further cultured for 24 h. Afterwards, the cells were fixed with methanol, blocked with 3% PBS-BSA for 30 min, incubated with a mixture of antibodies against CBS and β-tubulin or β-actin for 1 h, washed three times with 1 x PBS, and incubated with plus and minus PLA probes for 1 h. Then, the cells were washed (3 x 5 min), incubated for 40 min with ligation mixture containing connector oligonucleotides, washed again, and incubated with an amplification mixture containing a fluorescently labeled DNA probe for 100 min. After a final wash, the samples were mounted, and the signal was analyzed using a Zeiss LSM 510 Meta confocal microscope with a Plan Neofluar 40_/1.3 oil objective. The following antibodies were used: human CBS (1:100 dilution, AP6959c, Abgent, San Diego, CA, USA), β-tubulin (1:1000 dilution, ab231082, Abcam, Cambridge, UK), and β-actin (1:250 dilution, ab6276, Abcam, Cambridge, UK).

### Western blot analysis

Cells were scraped into 10 mmol/L Tris-HCl, pH 7.5, 1 mmol/L phenylmethyl sulfonyl fluoride (PMSF, Serva, Heidelberg, Germany) and protease inhibitor cocktail tablets (Complete EDTA-free, Roche Diagnostics, Indianapolis, IN, USA), as described previously (30), and centrifuged for 5 min at 3000 x g at 4°C. Pellet was re-suspended in Tris-buffer containing the 50 µmol/L CHAPS (3-[(3-cholamidopropyl) dimethylammonio] 1-propanesulfonate, Sigma-Aldrich, St. Louis, MO, USA) and then incubated for 30 min at 4°C. Lysate was centrifuged for 15 min at 10 000 x g at 4°C. Protein concentration was determined by the Modified Lowry Protein Assay Kit (Thermo Fisher Scientific, Waltham, MA, USA). Fifteen to forty micrograms of protein extract from each sample was separated by electrophoresis on 4-20% gradient SDS polyacrylamide gels. Afterwards, the proteins were transferred to the Hybond PVDF blotting membrane (GE Healthcare, Life Sciences, Chicago, IL, USA) using semidry blotting (Owl, Irvine, CA, USA). The membranes were blocked in 5% non-fat dry milk in TBS-T (Tris-buffered saline with Tween-20) overnight at 4°C and then incubated for 1 h with the primary antibody to β-actin (1:5000 dilution, ab6276, Abcam, Cambridge, UK) or β-tubulin (1:1000, ab108348, Abcam, Cambridge, UK), or the membranes were blocked in 5% non-fat dry milk in TBS-T or 5% BSA in TBS-T for 1 h at room temperature and then incubated overnight at 4°C with the appropriate primary antibodies to CBS (1:1000 dilution, ab144600, ab135626, Abcam, Cambridge, UK; 1:1000 ABIN1513863, Antibodies Online, Aachen, Germany; 1:1000 AP6959c, Abgent, San Diego, CA, USA), CSE (1:1000, ab136604, Abcam, Cambridge, UK), and MPST (1:500, ab154514, Abcam, Cambridge, UK). After washing, the membranes were incubated with secondary antibodies to mouse (secondary goat anti-mouse antibody, 1:10 000 dilution, ab6789, Abcam, Cambridge, UK) or rabbit (secondary goat anti-rabbit antibody, 1:10 000 dilution, ab97200, Abcam, Cambridge, UK) IgG conjugated to horseradish peroxidase for 1 h at room temperature. For visualization, a chemiluminescence detection system (Luminata™ Crescendo Western HRP Substrate, Millipore, Burlington, Mass., USA) was used. Each membrane was digitally captured using an imaging system (C-DiGit, LI-COR).

### Immunoprecipitation

The appropriate antibody was incubated with 60 µL washed magnetic beads, as described previously (31) (Dynabeads M-280), and coated with M-280 sheep anti-rabbit IgG (Invitrogen Dynal AS, Oslo, Norway) overnight at 4°C on a rotator (VWR International, Radnor, PA, USA). The beads with an attached antibody were washed (twice, 200 µL) with phosphate-buffered saline (PBS) with BSA. The proteins were immunoprecipitated from 0.5 mg of detergent-extracted total protein by incubation for 4 h at 4°C with antibody-bound beads. The bead complexes were washed four times with PTA solution (145 mmol/L NaCl, 10 mmol/L NaH2PO4, 10 mmol/L sodium azide, and 0.5% Tween020, pH 7.0). Immunoprecipitated proteins were then extracted with 60 µL of 2x Laemmli sample buffer (Bio-Rad, Hercules, CA, USA) and boiled *for 5 min.*

### Detection of H_2_S by H_2_S fluorescent probe, P3

The cells were plated on black 24-well plates or on glass coverslips. Following treatment with the 10 µmol/L GYY4137, the cells were incubated with 10 µmol/L of H_2_S Fluorescent Probe, P3 (Calbiochem, San Diego, CA, USA) for 30-60 min at 37°C in a water-saturated atmosphere with 5% CO_2_. Afterwards, the cells were twice washed with 500 µL of PBS. Fluorescence was measured on a Synergy H1 Reader (BioTek, Bad Friedrichshall, GE) at excitation 375 nm and emission 505 nm. The results were expressed as the arbitrary units of fluorescence. The fluorescent signal in the cells on the coverslips was visualized by a Zeiss Axiolab 5 FL microscope (Zeiss, Jena, Germany). The ZEN2 software was used to process the images and to evaluate the resultant pictures (Zeiss, Jena, Germany).

### Proliferation assay

The relative viability of the cells was determined by the CellTiter-Glo™Luminescent Cell Viability Assay (Promega Corporation, Madison, WI, USA) on a 96-well plate using 3000 cells per well and evaluated by the LumiStar GALAXY reader (BMG Labtechnologies, Germany) 4 days after plating and treatment with GYY4137.

### Cell migration assay

Fifty thousand DLD1, DLDx, DLD-NC, and DLD-PC cells per well were plated on ImageLock 96-well plates (Essen BioScience, Ann Arbor, MI, USA) and left to adhere for 24 h. Confluent monolayers were then wounded with a wound needle (IncuCyteWoundMaker, Essen BioScience, Ann Arbor, MI, USA), washed twice, and supplemented with a fresh culture medium or fresh culture medium with GYY4137. Images were taken every 2 h for the next 24 h via the IncuCyte ZOOM™ kinetic imaging system (Essen BioScience, Ann Arbor, MI, USA). Cell migration was evaluated by the IncuCyte ZOOM™ 2016A software (Essen BioScience, Ann Arbor, MI, USA) based on the relative wound density measurements.

### *In vivo* experiments

SCID/bg mice (male, 17 weeks old at the beginning of experiment) were bilaterally subcutaneously injected by 1x10^6^ DLD1, DLDx, DLD-PC, or DLD-NC cells resuspended in 100 µL of serum-free cultivation medium. Visible xenografts developed after 4 days. The mice were then randomly divided into either a treatment group (intraperitoneal injection of 20 mg/kg GYY4137 diluted in saline, given daily) or a control group (saline). Each group contained 13-22 tumors. Xenografts were measured every 3 days with a caliper, and the tumor volume (V) was calculated according to the formula: V=(length×width^×^2)/2, the width being the greatest transverse diameter and length the greatest longitudinal diameter. The mice were kept on standard pelleted food and water ad libitum, monitored daily for weight loss or other signs of possible toxic effects of the drug. At the experimental endpoints (after 14 days of therapy), the mice were sacrificed. The xenografts were resected, weighed, and cryopreserved at -80°C for further experiments or stored in buffered formalin.

#### Ethics approval

The study was conducted according to the guidelines of the Declaration of Helsinki and approved by the Institutional Ethic Committee and the national competence authority—State Veterinary and Food Administration of the Slovak Republic (project registration No. Ro-2032-3/2020-220)—in compliance with Directive 2010/63/EU and Regulation 377/2012 on the protection of animals used for scientific purposes. The project was conducted in the approved animal facility (license No. SK UCH 02017).

### *In silico* analysis

The Tumor online Prognostic analysis Platform (ToPP) (32), which collects multiomics and clinical data from 55 types of tumor datasets from The Cancer Genome Atlas (TCGA), International Cancer Genome Consortium (ICGC), and the Clinical Proteomic Tumor Analysis Consortium (CPTAC), was used for CBS gene expression analysis. The tumor versus normal expression and overall survival were analyzed using the TCGA-READ and TCGA-COAD datasets.

### Statistical analysis

The results are presented as mean ± S.E.M. Each value represents an average of at least three wells from at least three independent cultivations of each type of cell. Statistical differences among the groups were determined by one-way ANOVA. Statistical significance was considered to be significant at * or + - p < 0.05, ** or ++p < 0.01, and *** or +++p < 0.001.

## Results

### CBS has a similar immunofluorescence pattern to β-tubulin and β-actin in variety of cells

**Figure 1:**
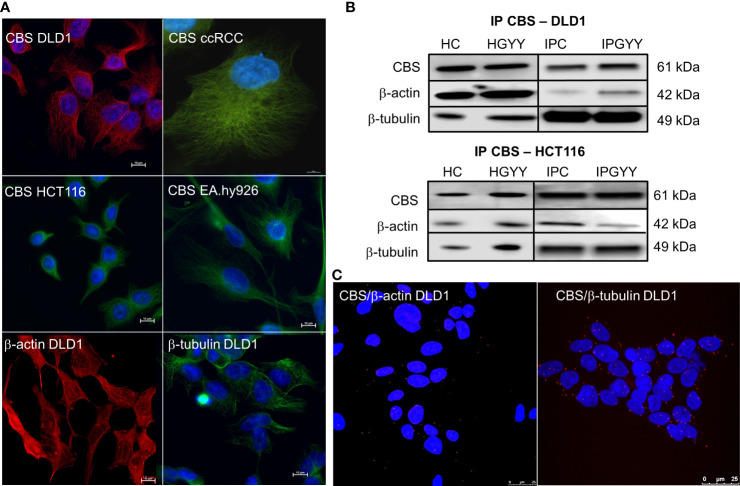
Cystathionine β-synthase (CBS) is bound to the cell cytoskeleton. Immunofluorescence with CBS revealed that this enzyme has a similar distribution pattern in various cells more so than β-actin and β-tubulin **(A)**. We performed immunoprecipitation of CBS with subsequent detection of β-actin and β-tubulin **(B)** in the control untreated DLD1 and/or HCT116 cells and cells treated with a slow sulfide donor GYY4137 (GYY, 10 µmol/L). We saw a signal of both these proteins in the immunoprecipitated sample. To verify the co-localization of CBS and β-actin/β-tubulin, we completed the proximity ligation assay in the DLD1 cells **(C)**, where we demonstrated a clear red signal showing co-localization of CBS with both cytoskeletal proteins. For **(A)**, scale bar represents 10 µm, for **(C)** 25 µm. IP, immunoprecipitation; CBS, cell proteins immunoprecipitated with CBS; ccRCC, stable cell line derived from clear cell renal cell carcinoma; DLD1, HCT116, human colorectal carcinoma cell line; EA.hy926, non-cancer endothelial cell line; HC, homogenate from untreated cells; HGYY, homogenate from cells treated with GYY4137; IPC, immunoprecipitation from untreated cells; IPGYY, immunoprecipitation from cells treated with GYY4137.

Immunofluorescence with the anti-CBS antibody revealed similar staining to β-tubulin and β-actin in variety of cells (**Figure 1A**). This staining pattern was visible in colorectal carcinoma cell lines DLD1 and HCT116, in the cell line derived from the clear cell renal cell carcinoma (ccRCC), and in the non-cancerous cell line derived from the EA.hy926 epithelial cells.

### Co-localization of CBS β-tubulin and/or β-actin

**Figure 2:**
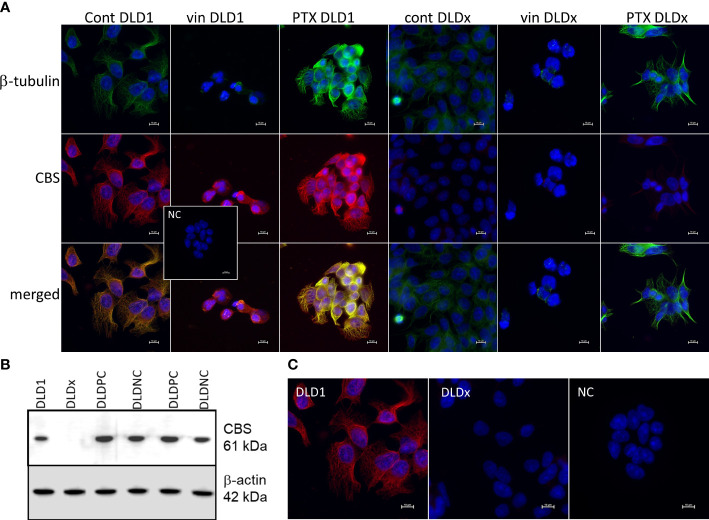
Preparation of DLD1/CBS_del cell line (DLDx) using CRISPR/Cas9 gene editing method and double immunofluorescence staining of β-tubulin (green) and cystathionine-β- synthase (CBS; red) in DLD1 and DLDx cells. Presence of CBS in the DLD1 and DLDx cells was tested by Western blot analysis **(B)** and immunofluorescence with CBS antibody **(C)**. We also prepared positive and negative CRISPR controls, as described in the Material and Methods. CBS was present in the positive (DLD-PC) and negative (DLD-NC) CRISPR controls. All these experiments support the fact that CBS was knocked out in the DLDx cells. Immunostaining was performed in control conditions and also after the treatment with vincristine (vin) and paclitaxel (PTX) for 24 h **(A)**. The nuclei were stained with DAPI (blue). Compared to the control untreated cells, in the presence of vin, the signals of β-tubulin and CBS were significantly suppressed, while in the presence of PTX, both signals were boosted. In the DLDx cells, tubulin net was detectable, although the CBS signal was not present. Inset shows the negative control, where both primary antibodies were omitted. Scale bar represents 10 µm.

We built a hypothesis that CBS might co-localize with β-tubulin and/or β-actin. To verify this hypothesis, we performed immunoprecipitation with the anti-CBS antibody and subsequent immunodetection with β-tubulin and β-actin antibodies in the DLD1 and HCT116 cells (**Figure 1B**). We observed a clear signal with both cytoskeletal antibodies and also in the presence of slow sulfide donor GYY4137 (GYY). Furthermore, we decided to show the co-localization of these proteins by a proximity ligation assay. In **Figure 1C**, the red dot signal shows the co-localization of CBS with both β-actin and β-tubulin in DLD1. To demonstrate the CBS interaction with β-tubulin, we performed double immunofluorescence staining with CBS (**Figure 2A**, red signal) and β-tubulin (green signal) in the control DLD1 group in a group of DLD1 cells treated with vincristine (which prevents polymerization of tubulin) or paclitaxel (which prevents depolymerization of tubulin). In the vincristine-treated DLD1 group, the β-tubulin signal diminished, similarly to the CBS signal. In the paclitaxel-treated DLD1 group, both the β-tubulin signal and CBS signal were much stronger compared to the untreated group (**Figure 2A**), thus proving the interaction of these two proteins. The specificity of the CBS and β-tubulin signals was verified by the negative control (**Figure 2A**, inset), where the primary antibodies were omitted. However, the β-tubulin net was detectable also in the DLDx cells (**Figure 2A**), although the CBS signal was not present. This would suggest that CBS is not crucial for the cytoskeleton, and it might just have a modulatory function.

### Functional role of CBS in colorectal carcinoma cells

**Figure 3:**
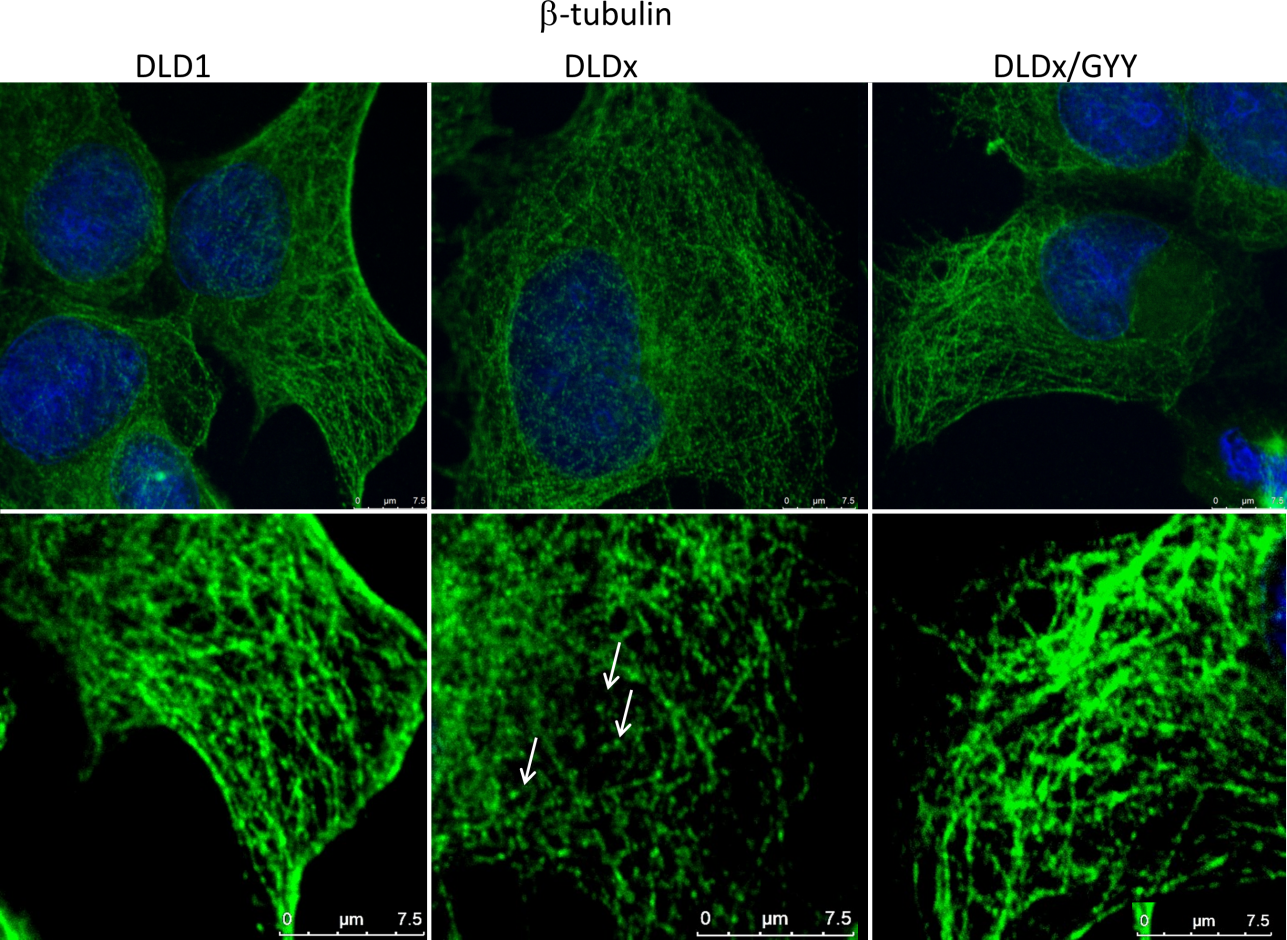
Effect of H_2_S on microtubule structure. We compared β-tubulin morphology (green) in the unmodified DLD1 cells, DLD1/CBS_del (DLDx) cell, and DLDx cells treated with slow sulfide donor GYY4137 (GYY). In the DLDx cells, we observed partial re-modulation of the β-tubulin skeleton (showed by arrows). After GYY treatment, this skeleton became the same as in the untreated cells. The nuclei were stained with DAPI. Scale bar represents 7.5 µm in both magnifications.

To study the functional role of CBS in colorectal carcinoma cells, we prepared a DLD1/CBS_del (DLDx) cell line with an inactive CBS gene through the CRISPR/Cas9 gene editing method. These clones were selected by puromycin selection, and CBS protein knockout was confirmed by WB analysis (**Figure 2B**). Absence of CBS in DLDx was demonstrated also by a proximity ligation assay with β-tubulin and CBS antibodies (not shown) and immunofluorescence with the CBS antibody (**Figure 2C**). We also prepared positive (DLD-PC) and negative (DLD-NC) CRISPR controls to eliminate possible false results due to CRISPR manipulations (**Figure 2B**). When comparing β-tubulin net in DLD1 and DLDx cells, we observed marked differences between these two groups of cells (**Figure 3**). The control group of DLD1 cells contained very well-defined microtubules with regular distribution in the cells (without accumulation in some cell parts). Individual microtubules showed homogenous structure. Compared to the control, the signal of β-tubulin in the DLDx cells was significantly weaker. The structure of microtubules was non-homogenous, with a presence of much brighter “spots” in their structure (**Figure 3**, arrows). In these cells, the microtubules showed accumulation mainly around the nuclei, and on the contrary, they were almost missing at the periphery of the cells, where their spot-like structure was very well evident. Application of GYY4137 led to the restoration of a homogeneous distribution of microtubules and to the homogeneity of individual microtubules (without spot-like structure, **Figure 3**).

### Determination of intracellular H_2_S

**Figure 4:**
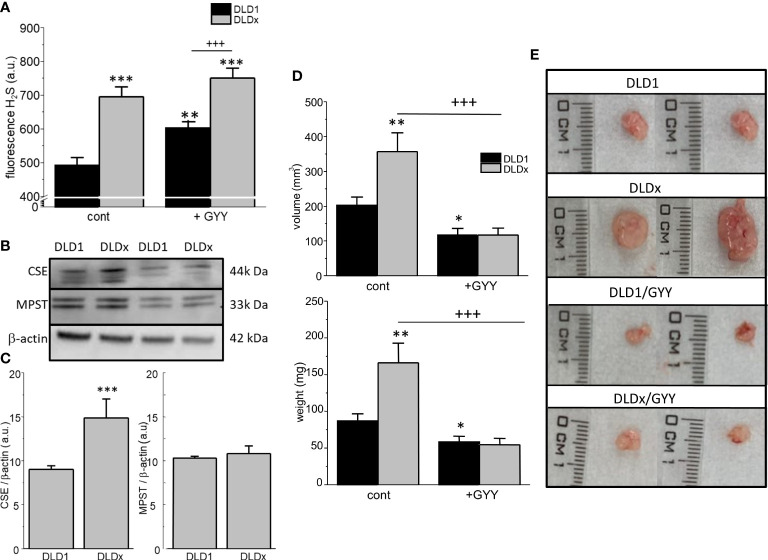
Determination of levels of intracellular H_2_S, other enzymes endogenously producing H_2_S, and xenograft formation in SCID/bg mice inoculated by DLD1 and DLDx cells. The intracellular level of H_2_S was significantly increased in the DLDx cells compared to DLD1, as determined by a fluorescent reader **(A)**, using H_2_S Fluorescent Probe, P3. Treatment of both cell lines with a slow sulfide donor, GYY4137 (GYY), also resulted in increased intracellular H_2_S levels compared to the untreated controls **(A)**. In the DLDx cells, the level of cystathionine-γ-lyase (CSE) was significantly higher compared to the DLD1 cells, while the level of 3-mercaptopyruvate sulfurtransferase (MPST) was not changed **(B, C)**. Immunodeficient SCID/bg mice were inoculated by either DLD1 or DLDx (with knocked-out CBS). On the fourth day, when small tumors started to become visible, the mice were divided into four groups. One of two DLD1 groups and one of two DLDx groups were simultaneously treated daily with GYY4137 (GYY). On termination day 14, the animals were sacrificed, and the xenograft’s volume (**D** upper graph, **E**) and weight of tumors (**D**, lower graph) were estimated. Xenografts from the DLDx cells were approximately twice as big in volume and weight compared to those from the DLD1 cells. When the mice were simultaneously treated with GYY, a significant decrease in volume and mass occurred in tumors from these cells. The volume and mass of tumors in the GYY-treated DLDx mice was significantly smaller **(D)**. Typical tumors from each group are shown in part **(E)**. Positive and negative CRISPR controls did not affect the size and/or volume of the tumors compared to the DLD1 group (not shown). The results in graphs **(A–C)** are displayed as mean ± S.E.M. and represent an average of 6-11 parallels from at least three independent cultivations. The results from the nude mice are displayed as mean ± S.E.M., and the number of tumors evaluated was n=13-22. Statistical significance * - p< 0.05, ** - p< 0.01, and *** represent p< 0.001 compared to the control DLD1 group. Statistical significance +++ represents p< 0.001 compared to the DLD1 group treated with GYY4137 **(A)** or to the DLDx group **(D)**.

The intracellular H_2_S level was determined in both cell lines by detecting fluorescence on a fluorescent reader (**Figure 4A**), namely, H_2_S Fluorescent Probe, P3. In the DLDx cells, the level of intracellular H_2_S was significantly higher compared to the DLD1 cells (**Figure 4A**). When both these cell lines were treated with a slow sulfide donor GYY4137 (GYY), an increase in the intracellular level of H_2_S compared to DLD1 was shown (**Figure 4A**). However, no changes in the intracellular H_2_S levels were observed when comparing the DLDx and DLDx/GYY groups (**Figure 4A**). Images from the fluorescence microscope revealed cytoplasmic localization of the P3 fluorescence signal (not shown).

### Detection of protein levels of other enzymes that endogenously produce H_2_S

Discrepancy between the increased level of intracellular H_2_S and the missing CBS protein in the DLDx versus DLD1 cells might be due to changes of other enzymes endogenously producing H_2_S–cystathionine-γ-lyase (CSE) and 3-mercaptopyruvate sulfurtransferase (MPST). The level of CSE protein was significantly increased in the DLDx cells compared to the DLD1 cells (**Figures 4B, C**), while the MPST protein level was not changed (**Figures 4B, C**).

### *In vivo* experiments on immunodeficient SCID/bg mice

Both the DLD1 and DLDx cells were injected subcutaneously into the immunodeficient SCID/bg mice. When small tumors started to be detectable, GYY4137 treatment was applied to two groups of mice (n = 13-22 tumors; **Figures 4D, E**). After 14 days, the mice were sacrificed, and the volume and the tumor’s weight were determined. Representative tumors of each group are shown in **Figure 4E**. The volume (**Figure 4D** upper graph, **E**) and weight (**Figure 4D** lower graph) of tumors were approximately twice as big in the mice inoculated with DLDx than in those with DLD1 cells. Interestingly, when the mice with DLDx tumors were simultaneously treated with GYY4137, the volume and weight of the tumors were significantly smaller and remained at values from the DLD1-inoculated mice (**Figure 4D**). Injection of positive and/or negative CRISPR cell lines (DLD-PC and DLD-NC) in the SCID/bg mice resulted in the same size of tumors as inoculation with DLD1 (not shown).

### Impact of CBS knockout on cell proliferation and migration

**Figure 5:**
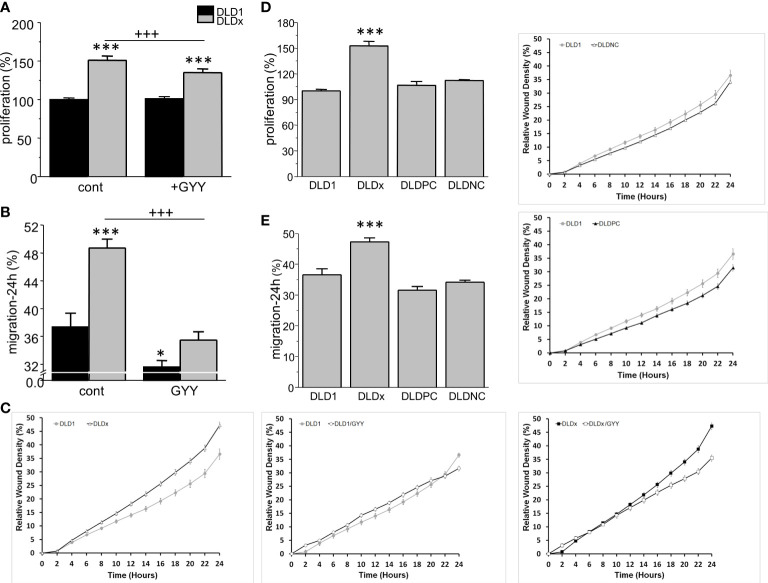
Differences in proliferation **(A)** and migration **(B, C)** between control and GYY4137 (GYY)-treated DLD1 and DLDx cells. Both proliferation and migration were higher in the DLDx cells compared to DLD1. After a treatment with GYY, proliferation was not affected in the DLD1 cells, but it was significantly decreased in the DLDx cells **(A)**. Migration was decreased in both DLD1 and DLDx after GYY treatment **(B)**. A typical result of migration curves is shown in **(C)**. Positive and negative CRISPR controls did not affect proliferation and/or migration **(D, E)**. The results are displayed as mean ± S.E.M. For proliferation, n=51-72, for migration, n=16-20 from three independent experiments, for positive and negative controls, n=16 from two independent experiments. Statistical significance * - p< 0.05 and *** represent p< 0.001 compared to the control DLD1 group. Statistical significance +++ represents p< 0.001 compared to the control DLDx group.

Proliferation and migration were also significantly higher in DLDx compared to DLD1 (**Figure 5**). Nevertheless, while proliferation was not affected by GYY4137 treatment in the DLD1 cells, a significant decrease was visible in the DLDx cells (**Figure 5A**). GYY4137 decreased migration in both the DLD1 and DLDx cells (**Figure 5B**). Control positive and negative CRISPR did not affect these processes (**Figures 5C–E**).

## Discussion

Using the cystathionine β-synthase antibody, we observed “fibred”-like immunofluorescent staining in the DLD1, HCT116, ccRCC and EA.hy926 cells, which suggests the binding of CBS to cytoskeletal proteins. Immunoprecipitation with the CBS antibody and a subsequent Western blot analysis and proximity ligation assay proved that CBS binds to β-tubulin and β-actin. Moreover, we compared the immunofluorescence of CBS and β-tubulin by using two microtubule targeting agents, both affecting β-tubulin–paclitaxel and vincristine [for a review, see (25, 33)]. Paclitaxel was shown to inhibit human cervical cancer cell division at low concentrations of the drug (0.25 mmol) and to block human cervical cancer cells in the G2/M phase (34). Blocking cells in the G2 phase was determined also in three colorectal carcinoma cell lines (35). Immunofluorescence of the DLD1 cells showed significantly increased staining of both β-tubulin and CBS in paclitaxel-treated cells compared to the control untreated ones. Additionally, when we used vincristine, a chemotherapeutic agent that prevents polymerization of β-tubulin, a very small signal of CBS and tubulin occurred compared to the controls. These results together with those previously discussed strongly suggest that CBS binds to cytoskeletal proteins in DLD1 cells.

It is obvious that the binding of CBS to cytoskeletal proteins might result in functional consequences. Changes in CBS binding might participate in re-organization of the cytoskeleton and potentially to altered tumorigenicity. There are several possibilities to explain the function of CBS on the cytoskeleton—either directly by stabilizing the cytoskeleton due to protein–protein interaction or indirectly through H_2_S. One possibility is that CBS stabilizes the cytoskeleton through binding to β-tubulin or β-actin. Another possible explanation lies in the local production of H_2_S. Although H_2_S can freely diffuse in the cell, it is probable that H_2_S first reacts with the targets in the close vicinity of its production that can ensure certain specificity of the H_2_S (as is the case of some other signaling molecules, e.g., calcium). Exogenous H_2_S is applied to the cells in much higher concentrations than is an endogenous production; therefore, it can diffuse to longer distances. This proposal is based on previous observations that cysteine residues in tubulin are actively involved in regulating ligand interactions and microtubule formation both *in vivo* and *in vitro*. These cysteine residues are sensitive markers in determining the conformation of tubulin. Tubulin dimer possesses 20 cysteine residues, from which twelve are localized in α-tubulin, and eight cysteines are in β-tubulin. Nevertheless, while the function of the β-tubulins in microtubule assembly is known, the function of β-tubulin isotypes in microtubule dynamics remains unknown (33). Cysteine oxidation is usually accompanied by loss of polymerization competence. Thus, it is not clear whether these modifications are only the result of oxidative stress or whether they also function as regulators under normal conditions. Certain cysteine residues of tubulin might regulate the dimer/microtubule equilibrium, and the thioredoxin system might, in turn, regulate this equilibrium (36).

Production of endogenous H_2_S is not performed solely by CBS but also by other enzymes, mainly CSE and MPST. CBS, CSE, and MPST are differently expressed in various tissues and organs, but their levels can also be up or downregulated under diverse conditions and in different tumor types. Different enzymes (or combinations of these enzymes) may play a role in H_2_S overproduction (for a review, see 37). We have found that CBS knocked out the DLDx cell line overexpressed CSE, which might be a compensatory mechanism for H_2_S production. Indeed, the level of cytosolic H_2_S was significantly higher in DLDx compared to the DLD1 cell line.

Expression of CBS differs in individual types of cancer and is definitely dependent on the cancer grade. The negative correlation of CBS expression with the pathologic parameters in hepatocellular carcinoma (HCC) indicates its potential as a prognostic marker in HCC (38). Moreover, levels of CBS were decreased by the higher grade of clear cell renal cell carcinoma (14). On the other hand, CBS, CSE, and MPST increased with the rise of malignant degrees in human bladder tissues and human UCB cell lines (39). Therefore, further studies on the role of CBS in tumorigenesis must consider tumor type and also grade.

**Figure 6:**
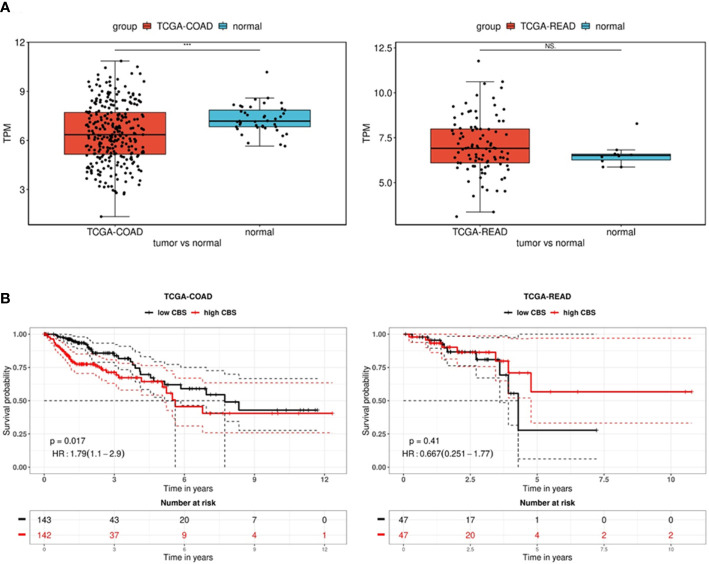
Analysis of CBS gene expression in the TCGA-COAD and TCGA-READ datasets. **(A)** Differential expression between tumor and normal tissue is presented as a boxplot for the two groups. The Wilcox test was performed to test whether there was a significant difference between the two groups. *** p < 0.001. The univariate analysis presented as a Kaplan–Meier (KM) survival plot **(B)** includes the p-value and hazard ratio with 95% confidence interval information.

In order to explore the expression and prognostic value of CBS in colon and rectal adenocarcinomas, we performed a comprehensive in silico analysis using the Tumor online Prognostic analysis Platform (ToPP) (32). **Figure 6** summarizes data from the ToPP, which were acquired after the selection of The Cancer Genome Atlas Colon adenocarcinoma (TCGA-COAD) and The Cancer Genome Atlas Rectum adenocarcinoma (TCGA-READ) datasets. A univariate analysis of CBS revealed a significantly decreased expression of the CBS gene in colon adenocarcinoma tumor tissue (n=285) (**Figure 6A** left) and non-significantly elevated expression of the CBS gene in rectum adenocarcinoma tumor tissue (n=94) (**Figure 6A** right).

Based on the expression status of CBS (CBS high/low), the overall survival presented as a Kaplan–Meier (KM) survival plot is depicted in **Figure 6B**. Kaplan–Meier curves show that although generally low CBS expression is associated with colon adenocarcinoma, reduced overall survival is significantly (p=0.017) associated with high CBS expression. In contrast, rectal adenocarcinomas show higher CBS expression compared to non-neoplastic tissue. However, low expression of CBS is associated with shorter overall survival, although it is statistically insignificant due to the lower number of evaluated patients (n=94). From the long-term survival, it is apparent that low CBS expression affects rectal carcinoma rather than colon adenocarcinoma.

Epigenetic factors (such as hypermethylation) should also be part of these studies. Downregulation of CBS through promoter methylation has been observed in multiple gastric cancer cell lines and four colon cancer cell lines (including HCT116) (40). More information is available about the relation of CBS expression in gastric cancer. The CpG island methylator phenotype (CIMP) is an epigenetic molecular subtype, which is observed in multiple malignancies and associated with the epigenetic silencing of tumor suppressors. A novel association between CBS epimutations and the CIMP subtype in gastric cancer was discovered, with *in vitro* models of CBS deficiency resulting in abnormal DNA methylation and inflammatory response (41).

To evaluate the physiological relevance of CBS in colorectal carcinoma cells, we used the CRISPR/Cas9 gene editing method (28, 29) to prepare the DLD1 cell line with knocked-out CBS (DLDx). Efficiency of the CRISPR was determined by Western blot and hybridization with the CBS antibody and by immunofluorescence and proximity ligation assay with the CBS and β-tubulin antibodies. To eliminate false results due to possible interference of the CRISPR technique, we also prepared a positive (DLDPC) and negative (DLDNC) CRISPR control. These controls normally expressed the CBS.

The β-tubulin immunostaining of DLDx revealed significant changes in cytoskeletal morphology compared to the unmodified DLD1 cells. In the DLDx cells, we observed partial destruction of the tubulin microfilaments, thus suggesting an important role of CBS and sulfide signaling, possibly through cysteine residues. It appears that CBS has a protective effect on microtubules. It was already shown that substitution of cysteine residues yields tubulin that is not capable of assembling into microtubules (42). However, reduced tubulin is polymerization competent, forming normal microtubules (36). Thus, a role for the cysteines in tubulin remains unresolved. Although there are several papers discussing the different roles of individual isotypes of β-tubulin in carcinogenesis (for a review, see 33), these isotypes have 85-95% similarity, and therefore, from the point of CBS binding, it is difficult to distinguish between them. Nevertheless, polymerization of β-tubulin depends on a wide variety of factors (e.g., phosphorylation and acetylation), and all these processes might be targeted by H_2_S produced by CBS. Further research is required to clarify this mechanism (which might be distinct from the production of H_2_S).

Moreover, we determined the levels of intracellular H_2_S in the DLD1 and DLDx cells. We found a significant increase of the intracellular H_2_S level in the DLDx cells compared to DLD1. This is in contrast to the eliminated CBS; therefore, we determined the protein levels of other endogenously producing enzymes, CSE and MPST. We observed a significant increase in the levels of CSE (and not of MPST), which might point to the compensatory effect of CSE in producing H_2_S. In various types of cancer, CBS and CSE expressed differently (16), so their mutual interaction and/or modulation might differ. Inhibition of CBS has shown anti-tumor activity, particularly in colon cancer, ovarian cancer, and breast cancer, whereas the consequence of CSE or 3MST inhibition remains largely unexplored in cancer cells (16). This observation uncovers a field for further studies.

The physiological relevance of the CBS/tubulin complex was studied by determining xenografts on immunodeficient SCID/bg mice and also proliferation and migration on DLD1 and DLDx cell lines. The xenografts obtained from the DLDx cells were significantly larger than those from the DLD1 cells. Parallel treatment of mice with GYY4137 resulted in the prevention of the DLDx tumor’s rapid growth, suggesting the involvement of H_2_S in this process. GYY4137 is a slow sulfide donor that releases H_2_S slowly and steadily, either in aqueous solution or administered to the animals, because of low toxicity (43). The effect of GYY4137 on tumor suppression has already been demonstrated on DLD1 and HepG2 cells (17, 18).

In cell cultures, DLDx also exhibited increased proliferation compared to DLD1, and GYY4137 significantly decreased this process in the DLDx cells but not in the DLD1 cells. This is in line with the observation of Zhang and co-workers (44), who demonstrated that endogenous overexpression of CBS and exogenous H_2_S could inhibit the proliferation and migration of colorectal carcinoma cells both *in vivo* and *in vitro*. Thus, it is apparent that expression of the CBS might be an important regulator of cell proliferation and migration. The mechanism behind how CBS, through cytoskeletal proteins, can affect tumor growth, proliferation, and migration needs to be studied in detail. As stated by Zuhra et al. (2), “even when the involvement of CBS in a given biological process is undisputable, it is often difficult to determine if the observed biological effects related to CBS are, in fact, due to upstream alterations (e.g., homocysteine accumulation due to CBS inhibition), downstream alterations (e.g., lack of production of cytoprotective cystathione or H_2_S after CBS inhibition) or global cellular changes (e.g., alterations in cellular glutathione levels and compensatory changes in redox balance)”. Likewise, protein–protein interaction should be taken into consideration regarding the function. It has already been demonstrated that silencing of the CBS in HCT116 cells decreases cell proliferation and attenuates HCT116 xenograft growth and vascularization in female Balb/c nude mice. These observations were performed either by silencing techniques or by CBS blockers, such as S-adenosyl-L-methionine (45) or aminooxyacetic acid (46), which did not allow the complete blockade of CBS. Using the CRISPR/Cas9 approach, one can ensure that CBS is permanently deleted. Furthermore, we used positive and negative CRISPR controls to eliminate possible false results caused by CRISPR technology.

## Conclusions

In summary, we have shown that in DLD1 colorectal carcinoma cells, CBS binds to the cytoskeletal proteins β-actin and β-tubulin, which has an impact on tumor growth, proliferation, and migration. When DLD1 cells with knocked-out CBS were inoculated into immunodeficient mice, larger tumors were obtained compared to the mice inoculated by the normal DLD1 cell line. Exogenous supplementation of H_2_S to the animals decreased the size of the tumors. When a slow sulfide donor, GYY4137, was added to the CBS knocked-out cell line, a decrease in proliferation and migration was shown.

## Data availability statement

The original contributions presented in the study are included in the article/supplementary material. Further inquiries can be directed to the corresponding author.

## Ethics statement

The study was conducted according to the guidelines of the Declaration of Helsinki, and approved by the Institutional Ethic Committee and by the national competence authority – State Veterinary and Food Administration of the Slovak Republic (project registration No. Ro-2032-3/2020-220) in compliance with the Directive 2010/63/EU and the Regulation 377/2012 on the protection of animals used for scientific purposes. Project was conducted in the approved animal facility (license No. SK UCH 02017). Written informed consent was obtained from the owners for the participation of their animals in this study.

## Author contributions

Conception and design: OK. Development of methodology: VL, IR, and MM. Acquisition of data: VL, BC, PB, IR, and KP. Data analysis and interpretation: VL, BC, PB, MM, and OK. Writing of the manuscript: VL, MM, and OK. In silico analysis: IR. Study supervision: OK. Funding acquisition: OK and VL. The authors have read and agreed to the published version of the manuscript.
